# Effects of critical illness on the functional status of children with a history of prematurity

**DOI:** 10.5935/0103-507X.20220429-en

**Published:** 2022

**Authors:** Millene Albeche Peduce, Vanessa Campes Dannenberg, Paula Maria Eidt Rovedder, Paulo Roberto Antonacci Carvalho

**Affiliations:** 1 Postgraduate Program in Child and Adolescent Health, Universidade Federal do Rio Grande do Sul - Porto Alegre (RS), Brazil.; 2 Physiotherapy School, Universidade Federal do Rio Grande do Sul- Porto Alegre (RS), Brazil.; 3 Pediatric Intensive Care Unit, Department of Pediatrics, Hospital de Clínicas de Porto Alegre, Universidade Federal do Rio Grande do Sul - Porto Alegre (RS), Brazil.

**Keywords:** Premature, Morbidity, Child, hospitalized, Intensive care units, pediatric, Functional status

## Abstract

**Objective:**

To evaluate the effects of critical illness on the functional status of children aged zero to 4 years with or without a history of prematurity after discharge from the pediatric intensive care unit.

**Methods:**

This was a secondary cross-sectional study nested in an observational cohort of survivors from a pediatric intensive care unit. Functional assessment was performed using the Functional Status Scale within 48 hours after discharge from the pediatric intensive care unit.

**Results:**

A total of 126 patients participated in the study, 75 of whom were premature, and 51 of whom were born at term. Comparing the baseline and functional status at pediatric intensive care unit discharge, both groups showed significant differences (p < 0.001). Preterm patients exhibited greater functional decline at discharge from the pediatric intensive care unit (61%). Among patients born at term, there was a significant correlation between the Pediatric Index of Mortality, duration of sedation, duration of mechanical ventilation and length of hospital stay with the functional outcomes (p = 0.05).

**Conclusion:**

Most patients showed a functional decline at discharge from the pediatric intensive care unit. Although preterm patients had a greater functional decline at discharge, sedation and mechanical ventilation duration influenced functional status among patients born at term.

## INTRODUCTION

Prematurity has been reported to be one of the most important comorbidities related to chronic diseases, recurrent consultations and pediatric hospital admissions.^(1)^

As a public health challenge, prematurity is associated with a primitive stage of lung development and a greater risk of delayed neuropsychomotor development and learning and/or behavioral difficulties in early childhood.^(1)^

Currently, the considerable survival of premature infants has brought new concerns to health professionals and researchers and highlighted the need for new health indicators for this population of survivors.^(2)^ Thus, the evaluation of the functional status of children with a history of prematurity after a critical illness has become an important resource for improving the quality of care of these patients.^(3)^

The Functional Status Scale (FSS) is one of the few instruments validated for the Brazilian population to measure the functionality of children hospitalized after critical illness.^(4)^ In addition, it stands out as a quantitative method that is fast and easy to apply, covering the age group of newborns up to 18 years.^(3)^

This study aims to evaluate the effects of critical illness on the functional status of patients with or without a history of prematurity who were discharged from the pediatric intensive care unit (ICU) through the FSS. In addition, we verified the relationship between the functional status of these children at discharge from the pediatric ICU and the Pediatric Index of Mortality (PIM2), ventilation time, sedation time and length of hospital stay.

## METHODS

This is a secondary cross-sectional study nested in an observational cohort of survivors from a tertiary pediatric ICU admitted from September 2016 to October 2017.

The data used in this study were extracted from the original study database previously submitted to the Research Ethics Committee of the *Hospital de Clínicas de Porto Alegre* under opinion 2,646,290. The informed consent form was signed by the guardians for permission to collect the data.

All clinical and surgical patients, except those with trauma and cardiac surgery, up to 4 years of age, were selected from the original study sample.^(5)^ Therefore, the present study had a sample of 126 patients: 75 with a history of prematurity and 51 full-term. Children with a hospitalization time < 24 hours, readmissions to the pediatric ICU for <48 hours, children in palliative care, children born at term with previous chronic conditions, patients transferred from the neonatal ICU and patients with a corrected age below 1 month of life were excluded from the study. Regarding dependence on technology, the use of technologies related to feeding (nasogastric tube, enteral tube and gastrostomy) and breathing (oxygen dependence, tracheostomy, non-invasive ventilation and invasive mechanical ventilatory support) was considered.

Two researchers performed data collection. The first researcher selected the patients, applied the consent form and collected information on functional status before admission. The same researcher collected information regarding the length of stay in the pediatric ICU from electronic medical records. The second researcher, blinded to the demographic and clinical information of the patients, evaluated the functional status of the survivors from the pediatric ICU in the period up to 48 hours after discharge from that unit.

The evaluation of functionality was performed by the FSS, which is composed of six domains: mental state (1), sensory functioning (2), communication (3), motor functioning (4), feeding (5) and respiratory status (6). For each domain, a score ranging from one to six is used, with one being considered good/adequate and six as very severe dysfunction. The total sum of the scores of the domains generates a score ranging from 6 to 30 points, with the following classification: 6 - 7, adequate; 8 - 9, slightly abnormal; 10 - 15, moderately abnormal; and 16 - 21, severely abnormal. The higher the score is, the greater the functional impairment of the patient will be.

The power to test two independent proportions was calculated using the Power and Sample Size for Health Researchers (PSS Health) *online* tool. A significance level of 5%, outcome proportions of 48% and 76%, and sample sizes of 51 and 75 were considered in the groups of full-term and premature births, respectively, with continuity correction being applied and reaching power of 86.3%.

### Statistical analysis

The quantitative variables were tested for normality using the Shapiro-Wilk test and are described as the median and interquartile range of 25 - 75, according to the distribution. The total sample was divided into two groups, according to gestational age (GA), preterm birth (GA < 37 weeks) and full-term birth, and the comparison between groups was performed using Pearson’s chi-square test, Fisher’s exact test, Yates’s continuity correction, or the Mann-Whitney U test. The Wilcoxon test was used to verify differences between the functional statuses at two-time points. The Spearman correlation test was used to assess the degree of correlation between clinical variables and functional status at discharge from the pediatric ICU.

Due to the sample size, for statistical analysis, patients classified as having a severely abnormal and very severely abnormal functional status were considered to be in the same group and were described as “severely/very severely abnormal”.

Patients who presented an increase in the total sum of the FSS scale domains sufficient to alter their functional condition were classified as having an altered functional outcome.

The analyses were performed using the Statistical Package for Social Sciences (SPSS) version 21.0 with a significance level of 5% (p ≤ 0.05).

## RESULTS

**Figure 1:**
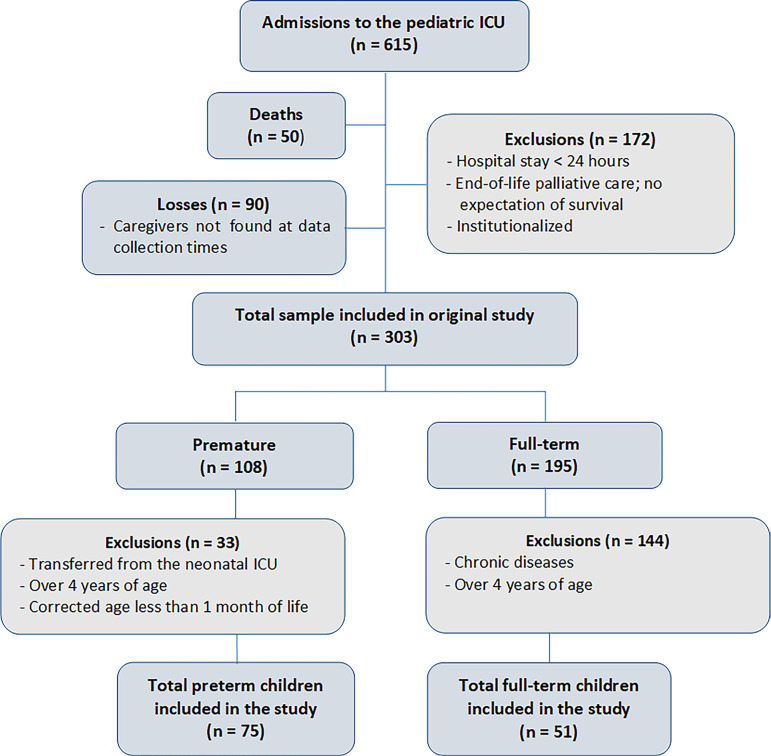
Study flowchart.

There were 615 admissions to the pediatric ICU during the collection period, and initially, 303 patients were selected (Figure 1). After applying the exclusion criteria, the total sample of the present study consisted of 126 patients comprising 75 preterm and 51 full-term.

ICU - intensive care unit.

The main reason for admission to the pediatric ICU was due to respiratory illnesses (58%), representing almost half of premature (48%) and 72% of full-term infants (Table 1).

**Table 1:** General characteristics of the sample and association between variables and groups (preterm and full-term children)

	Total (n = 126)	Premature (n = 75)	Full-term (n = 51)	p value
Previous characteristics				
Male sex	83 (66)	50 (67)	33 (65)	0.97^*^
Corrected age (months)¶	5 (2 - 15)	8 (2 - 21)	4 (1 - 7)	0.004†
Birth weight (g)				
1.000 - 1.500	20 (16)	20 (27)	0	
1.501 - 2.500	40 (32)	36 (48)	4 (8)	< 0.001‡
< 2.500	66 (52)	19 (25)	47 (92)	
Complications in childbirth	58 (46)	42 (57)	16 (31)	0.07^*^
Use of continuous medication	51 (40)	50 (67)	1 (2)	< 0.001^*^
Technology at admission	44 (35)	44 (59)	0	< 0.001^*^
None	82 (65)	31 (41)	0	
Food	12 (9.5)	12 (16)	0	< 0.001§
Respiratory	2 (1.6)	2 (3)	0	
More than one technology	30 (24)	30 (40)	0	
Previous hospitalization	100 (79)	71 (95)	29 (57)	< 0.001^*^
Main caregiver				
Mother	103 (82)	58 (78)	45 (88)	
Father	9 (7)	4 (5)	5 (10)	0.08§
Grandmother	11 (9)	11 (15)	0	
Other	2 (1,6)	1 (1)	1 (2)	
Caregiver educational level				
Incomplete elementary school	35 (28)	20 (27)	15 (30)	
Complete elementary school/Incomplete secondary school	39 (31)	23 (32)	16 (32)	0.97§
Complete high school/Incomplete higher education	42 (33)	26 (36)	16 (32)	
Complete higher education	7 (6)	4 (6)	3 (6)	
Clinical factors				
PIM2	0.99 (0.27 - 4.24)	1.37 (0.4 - 9.6)	0.65 (0.16 - 2)	0.02†
Reason for admission				
Neurologic	7 (5)	6 (8)	1 (2)	0.03§
Respiratory	73 (58)	36 (48)	37 (72)	
Surgical procedure	32 (25)	24 (32)	8 (16)	
Other	6 (5)	9 (12)	5 (9)	
Use of sedatives	95 (75)	60 (80)	35 (69)	0.21^*^
Sedation time (days)	2 (0 - 6)	2 (1 - 6)	1 (0 - 5)	0.20†
Neuromuscular blocker	27 (21)	17 (23)	10 (20)	0.85^*^
Adverse event/complication	46 (36)	32 (43)	14 (28)	0.12^*^
Use of IMV	61 (48)	38 (51)	23 (45)	0,66^*^
Time of IMV (days)	0 (0 - 6)	1 (0 - 6)	0 (0 - 6)	0,49†
Length of hospital stay	7 (4 - 10)	7 (4 - 10)	6 (4 - 10)	0.14†
Functional decline at discharge	91 (72)	61 (81)	30 (59)	0.01^*^
Use of technology at discharge	105 (84)	65 (87)	40 (78)	0.23^*^
Type of technology				
None	20 (16)	9 (12)	11 (22)	0.01‡
Food	14 (11)	9 (12)	5 (9)	
Respiratory	19 (15)	6 (8)	13 (25)	
More than one technology	73 (58)	51 (68)	22 (43)	

PIM2 - Pediatric Index of Mortality; IMV - invasive mechanical ventilation. Statistical significance = p <0.05.

* Yates continuity correction; † Mann-Whitney U test; ‡ Pearson’s chi-square test; § Fisher’s exact test; ¶ Corrected age calculation performed for patients born preterm who had a chronological age ≤ 24 months at the time of admission. Values are described as n (%) and median and interquartile range (25 -75).

Comparing preterm and full-term children, significant differences were observed regarding age at admission, birth weight, use of technology prior to admission and previous hospitalizations (p < 0.05). Premature patients also had higher disease severity at admission, represented by PIM2 (p = 0.02).

At the time of discharge from the pediatric ICU, the number of technology-dependent patients increased significantly (p < 0.001). The percentage of children with a history of prematurity who required technological support at the time of discharge from the pediatric ICU was 87%, representing an increase of 28% (p < 0.001). An even greater increase in the need for technological support at discharge from the pediatric ICU occurred in patients born at term (78%) because, prior to admission, no patient born at term needed technological support. At hospital discharge, it was found that in the group of full-term patients, 43% used more than one type of technology (Table 1).

**Figure 2:**
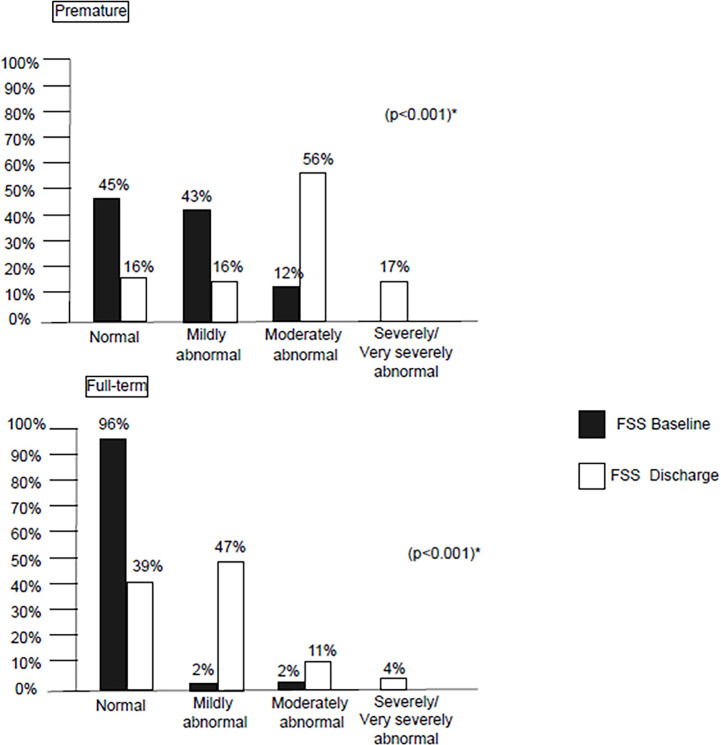
Comparison of the functional *status* of preterm and full-term patients at baseline and discharge from the pediatric intensive care unit according to the Functional Status Scale.

Regarding functional status, 72% of all patients, comprising 81% of premature children and 59% of full-term, exhibited functional decline at the pediatric ICU discharge (p = 0.01). Both groups showed significant functional changes at discharge (p < 0.001) (Figure 2). Forty-two (56%) children born preterm had moderate changes in functional status, and 13 (17%) had severe/very severe functional alterations at discharge from the pediatric ICU, representing a 61% increase in patients with moderate to very severe alterations compared to admission. Regarding the group of full-term children, there was also an increase in the number of patients with moderate to very severe functional changes, which, although to a lesser extent (12%), was also significant (p < 0.001).

FSS - Functional Status Scale. * Wilcoxon test.

In the comparison of functional status, there were significant differences between preterm patients and those born at term (p < 0.001) (Table 2). At admission, 55% of preterm patients already had mild or moderate changes in functional status, while only 4% of full-term patients were classified as having some functional change. At discharge from the pediatric ICU, the number of patients with functional changes increased significantly in both groups (p < 0.001); 17% of children born preterm and 4% of those born full-term had severe/very severe functional changes.

**Table 2:** Comparison of the functional status of children born preterm and full-term before and after critical illness

	Preterm (n = 75)	Full term (n = 51)	p value^*^
Baseline functional status (admission)			
Normal	33 (45)	49 (96)	<0.001
Mildly abnormal	32 (43)	1 (2)	
Moderately abnormal	9 (12)	1 (2)	
Severely/Very severely abnormal	0	0	
Functional status at discharge from the pediatric ICU			
Normal	8 (11)	20 (39)	< 0.001
Mildly abnormal	12 (16)	24 (47)	
Moderately abnormal	42 (56)	5 (11)	
Severely/Very severely abnormal	13 (17)	2 (4)	

ICU - intensive care unit.

* Pearson’s chi-square test. Values are described as n (%).

In the correlation analysis of the clinical variables with the functional outcome, a significant (p = 0.04) but weak (ρ = 0.23) correlation was observed between age at admission and the functional outcome of preterm patients (Table 3). In the group of patients born at term, there was a significant correlation between PIM2, duration of sedation, duration of mechanical ventilation (MV) and length of hospital stay with functional outcomes (p = 0.05). The time of sedation and time of MV exhibited moderate correlation with the functional outcome of patients born at term (ρ = 0.48 and ρ = 0.54, respectively), indicating that the longer the use of sedatives and MV during pediatric ICU hospitalization, the greater the functional impairment for full-term patients seems to be.

**Table 3:** Correlation between clinical variables and functional outcome at discharge from the pediatric intensive care unit

Variables	FSS functional outcome			
	Premature		Full-term	
	ρ	ρ	ρ	ρ
Age (months)^*^	0.23	0.04	-0.14	0.30
PIM2	-0.11	0.34	0.31	0.02
Sedation time (days)	0.12	0.27	0.48	< 0.001
Time of IMV (days)	0.10	0.38	0.54	< 0.001
Length of hospital stay	0.13	0.26	0.37	0.006

FSS - Functional Status Scale; ρ - Spearman’s rho; PIM2 - Pediatric Index of Mortality; IMV - invasive mechanical ventilation.

* Corrected age calculation performed up to 2 years of age for preterm patients. Statistical significance = p < 0.05.

## DISCUSSION

In the present study, the rate of total functional decline, considering both groups of patients, was 72%. In addition, both groups showed significant changes in functional status at the time of discharge from the pediatric ICU (p < 0.001). However, preterm patients had a higher percentage of functional decline (81%) than full-term children (59%) (p = 0.01). No other studies were found in the literature comparing the functional status of patients with a history of prematurity with those born at term after critical illness or considering measures of functional status at baseline and at the time of discharge from the pediatric ICU. It is believed that these findings may provide background information for future studies and thus contribute to a better understanding of the functional outcomes of pediatric patients surviving critical diseases.

Previous studies conducted in the same pediatric ICU reported rates of functional decline similar to those found in this study. Alievi et al. evaluated the impact of admission to the pediatric ICU on cognitive and functional performance in 433 children by applying the Pediatric Overall Performance Category (POPC), and Pediatric Cerebral Performance Category (PCPC) scales at admission and discharge. In this study, the authors found that, at discharge, 60% of children had some degree of cognitive morbidity, and 86% had some degree of functional morbidity.^(6)^ Another study that evaluated the functionality through the FSS of 50 children after discharge from the pediatric ICU found some degree of alteration in the FSS domains in 82% of patients.^(7)^ Comparing the prevalence of functional decline found in the present study with data reported in studies conducted in pediatric ICUs in other countries, the prevalence of functional decline in our unit was higher. The prevalence of functional decline at discharge reported by the international literature ranges from 5.2 to 36%.^(8-10)^ It is believed that this difference in the prevalence of functional decline among the different studies can be explained by the evaluation instrument used and/or by the characteristics and resources available in each pediatric ICU.^(11)^

In this study, a higher percentage of functional decline (81%) was identified in patients with a history of prematurity compared to those born at term (59%), and there was a more evident increase (61%) of moderate to very severe functional changes in the group of children born prematurely compared with those full-term born (12%). This higher rate of functional decline may be explained by the fact that, historically, preterm patients have a higher rate of hospitalizations during the first year of life than patients born at term.^(11)^ In addition, children born preterm tend to show significant differences in growth and neurodevelopment compared to those born at term.^(12,13)^ In this sense, a study conducted in Sweden, which followed a cohort of premature for 45 years, reported that low GI at birth was related to increased mortality from childhood to adulthood.^(14)^ Similarly, Blencowe et al. provided evidence on the origins of chronic diseases in early life supported by the theory of the origins of development.^(12)^ Therefore, it is believed that in our study, the highest percentage of functional decline found in the group of children with a history of prematurity can be argued by the evidence that prematurity is considered a risk factor for worse health conditions.^(14)^

In the analysis of the influence of clinical outcomes on functional variables, it was found that at the time of discharge, the functional status of children born full-term showed a moderate correlation with the duration of sedation (ρ = 0.48; p < 0.001) and duration of use of MV (ρ = 0.54; p < 0.001). Thus, this finding shows that the longer the use of sedatives and MV during pediatric ICU hospitalization, the greater the functional impairment for full-term patients seems to be. In the literature, longer periods of MV are associated with greater chances of functional decline at pediatric ICU discharge.^(6,8,9)^ It is known that MV support is essential to ensure survival in situations inherent to pediatric intensive care and that, to be used, the child is usually under the effect of sedative and analgesic medications.^(15)^ Thus, it is believed that the correlation between the use of MV and sedatives with greater functional decline found in full-term patients may be related to the loss of muscle strength secondary to periods of greater immobilization and bed restraint. The effects of prolonged immobility, as one of the main causes of acquired muscle weakness, have already been reported in adult intensive care patients.^(16)^ However, in pediatrics, the effects of muscle mass loss are unclear due to the difficulty in implementing standardized methods to assess muscle strength in children.^(17)^ Valla et al.^(17)^, in one of the few studies that evaluated muscle strength in critically ill pediatric patients, found that in 17 children subjected to MV, the thickness of the quadriceps femoris muscle decreased considerably by 9.8% after 5 days of hospitalization. These findings highlight the importance of applying early mobilization protocols, according to the clinical and hemodynamic stabilization of the patient, to avoid functional impairment, as has been discussed in previous studies.^(18)^

Respiratory diseases, in general, are one of the main factors associated with hospitalization in the first 6 years of life.^(19)^ Thus, we believe that the significant correlation between functional decline, use of MV and sedatives found in our study may also be related to the profile of patients, since most admissions to this pediatric ICU occurred due to respiratory illnesses. Previous studies conducted in the same pediatric ICU also reported respiratory illnesses (40% and 43%) as the most frequent causes of admission.^(5,7)^

In this study, the clinical outcomes evaluated (duration of MV, sedation and hospitalization) did not show a significant relationship with functional status in the patients with a history of prematurity. It is believed that this result may be explained by the fact that more than half of preterm patients (57%) needed some technology at admission and, because of that, had already some previous functional change. In addition to presenting significant differences in age at admission, birth weight and previous hospital admissions (p < 0.05) compared to patients born at term, preterm patients also showed greater disease severity at admission (p = 0.02). These factors are believed to complement each other because factors such as age at first hospitalization, number of previous hospitalizations, disease severity and socioenvironmental conditions are considered risk factors for repeated hospitalizations.^(19)^ Moreover, children born prematurely and with low birth weight have an odds ratio of hospitalization almost three times higher than those born at term and with adequate weight during the first year of life.^(19)^

The present study has some limitations, such as that it is a secondary data analysis, which limited the size of this sample. In addition, the data refer to patients from a single center, preventing the generalization of the findings. However, there are only two Brazilian studies investigating the functional status of patients after discharge from the pediatric ICU and considering baseline functional status measurements.^(5^.^7)^

In addition, no other studies were found comparing the functional status of patients with a history of prematurity with those patients born full-term after the critical illness episode.

## CONCLUSION

Most of the studied patients showed a functional decline at the time of discharge from the pediatric intensive care unit. The functional status of patients born at term was influenced by the duration of sedation and the duration of mechanical ventilation, and this was not observed among children with a history of prematurity. Regarding the previous dependence on health technologies, patients born at term did not require technological support prior to admission, unlike those born prematurely. In general, at the time of discharge from the pediatric intensive care unit, the number of technology-dependent patients significantly increased.
